# News and Notes

**Published:** 1903-08

**Authors:** 


### NEWS AND NOTES.

Dr. George H. Fox, Professor of Dermatology in Columbia University, has been appointed dermatologist to the New York State Department of Health.

---

John Perrier, M.D., Professor of Theory and Practice of Medicine in the Cleveland College of Physicians and Surgeons, died from typhoid fever at his home in Cleveland, after an illness of four months, aged sixty.

---

Dr. Lewis S. McMurtry, of Louisville, delivered the address on surgery before the South Carolina Medical Association at its recent meeting in Charleston. He chose for his subject fibroid tumors of the uterus and their complications.

---

Edward Charles Wendt, M.D., a member of the editorial staff of the *Medical Record* for several years, and an authority on sanitation and public health, committed suicide, while temporarily insane from illness, by shooting himself. He was residing in Paris at the time, and had been making exhaustive studies of the methods of sanitation in Europe.

---

At the annual meeting of the American Gastro-enterological Association the following officers were elected for the ensuing year: President, Dr. S. J. Meltzer, of New York; First Vice-President, Dr. Fenton B. Turck, of Chicago; Second Vice-President, Dr. William Gerry Morgan, of Washington, D. C.; Secretary and Treasurer, Dr. Charles D. Aaron, of Detroit, Mich.

---

The officers of the American Proctologic Society recently elected are: President, Dr. William M. Beach, of Pittsburg;

Vice-President, Dr. Leon Strauss, of St. Louis; Secretary-Treasurer, Dr. A. Bennett Cooke, of Nashville; Executive Council, Dr. Samuel T. Earle, of Baltimore (retiring president), chairman; Dr. George B. Evans, of Dayton, O.; Dr. John L. Jelks, of Memphis, Tenn.

---

AT the Fourteenth International Medical Congress, Madrid, the prize of $1,000, offered in 1897 by the City of Moscow, to be awarded every three years for the work which has contributed most to the progress of medical science, was given to Dr. Elie Metchnikoff, professor in the Pasteur Institute, Paris. The prize offered by the Thirteenth International Congress, held in Paris in 1900, was awarded to Dr. B. Grassi, professor of comparative anatomy at Rome.

---

AT the meeting of the American Gynecological Association held in Washington, the following officers were elected for the ensuing year: President, Dr. Edward W. Reynolds, of Boston; First Vice-President, Dr. J. Whitridge Williams, of Baltimore; Second Vice-President, Dr. E. P. Davis, of Philadelphia; Secretary, Dr. J. Riddle Goffe, of New York; Treasurer, Dr. J. Montgomery Baldy, of Philadelphia; member of Council, Dr. J. E. Janvrin, of New York. The next meeting will be held in Boston.

---

AT the sixth annual meeting of the Association of Medical Librarians the following officers were elected: President, Dr. William Osler, of Baltimore; Vice-President, Dr. Abraham Jacobi, of New York; Secretary, Mr. Albert T. Huntington, of Brooklyn; Treasurer, Dr. George D. Hersey, of Providence; Executive Committee, Mr. John S. Browne, of New York; Mr. Charles P. Fisher of Philadelphia, and Dr. James M. Winfield, of Brooklyn. The meeting adjourned to meet in June, 1904, at Atlantic City, N. J.

WHAT promised to be a useful and honorable career was suddenly cut short by the assassination at Puerto Cortez, Honduras, of Dr. Albert Ferrari, a recent graduate of the Medico-Chirurgical College of Philadelphia. Dr. Ferrari was a native of Honduras, had received his medical education here and had especially prepared himself under Prof. L. Webster Fox for the treatment of diseases of the eye, so common in the land of his birth. Arrived at home he visited his cousin, chief of police of Puerto Cortez. That official had become involved in a quarrel with an officer of the army and had challenged him to a duel. Instead of personally appearing at the place of meeting the officer sent a file of soldiers, whose fire, by a freak of Honduras marksmanship, killed Dr. Ferrari, who was present in his professional capacity. The victim's friends and associates in this city are filled with regret and grief at the lamentable termination of a life which had just crossed the threshold of professional existence.

---

AT the recent meeting of the Association of Military Surgeons of the United States, the Enno Sander prize, a $100 gold medal, for an essay on the differential diagnosis of typhoid fever in its early stages, was awarded to Major Frederick Smith, of Wiltshire, England. The second prize, given because a second essay was considered nearly as good as the first, consisting of life-membership in the association, was awarded to William C. Rucker, U. S. P. H. and M.-H. S., of Boston. A large number of papers were read during the meeting. Surgeon-General Johann von Mikulicz-Radecki, chief medical officer of the Sixth Prussian Army Corps, was present at the meeting. The following officers were elected for the ensuing year: President, Medical Director John C. Wise, U.S.N.; First Vice-President, Surgeon-General Walter Wyman, of the U. S. P. H. and M.-H. S.; Second Vice-President, Major Albert H. Briggs, Buffalo, N. Y.; Third Vice-President, Surgeon-General Robert M. O'Reilly, U. S. A.; Secretary, Major James Evelyn Pilcher, U. S. A.; Treasurer, Major H. A. Arnold, Ardmore, Pa.

